# High EGFR protein expression and exon 9 *PIK3CA* mutations are independent prognostic factors in triple negative breast cancers

**DOI:** 10.1186/s12885-015-1977-3

**Published:** 2015-12-18

**Authors:** William Jacot, Caroline Mollevi, Frédéric Fina, Evelyne Lopez-Crapez, Pierre-Marie Martin, Pierre-Emmanuel Colombo, Frédéric Bibeau, Gilles Romieu, Pierre-Jean Lamy

**Affiliations:** Department of Medical Oncology, Montpellier Cancer Institute Val d’Aurelle, 208, rue des Apothicaires, Montpellier, F-34298 France; Translationnal Research Unit, Montpellier Cancer Institute Val d’Aurelle, 208, rue des Apothicaires, Montpellier, F-34298 France; Biostatistical Research Unit, Montpellier Cancer Institute Val d’Aurelle, 208, rue des Apothicaires, Montpellier, F-34298 France; Department of Oncobiology, Assistance Publique – Hôpitaux de Marseille, Boulevard Pierre Dramard, Marseille, F-13916 France; Department of Surgical Oncology, Montpellier Cancer Institute Val d’Aurelle, 208, rue des Apothicaires, Montpellier, F-34298 France; Department of Pathology, Montpellier Cancer Institute Val d’Aurelle, 208, rue des Apothicaires, Montpellier, F-34298 France; Department of Oncogenetics, Montpellier Cancer Institute Val d’Aurelle, 208, rue des Apothicaires, Montpellier, F-34298 France; Biological Ressources Center, Montpellier Cancer Institute Val d’Aurelle, 208, rue des Apothicaires, F-34298 Montpellier, France

**Keywords:** Triple negative, Breast cancer, EGFR, Gene amplification, PI3K, PTEN

## Abstract

**Background:**

Triple negative breast cancers (TNBC) are a more aggressive subset of breast cancer. A better understanding of its biology could allow the rational development of targeted therapies.

**Methods:**

We extensively analyzed the EGFR/PI3K/PTEN axis in a large, homogeneous population of TNBC to help defining the putative role of anti-EGFR and -PI3K targeted therapies in this setting. *EGFR* gene amplification, EGFR protein expression, *PIK3CA* and *PTEN* gene alterations (two members of EGFR downstream pathways) and their clinicopathological and prognostic implications were analyzed in 204 TNBC samples from European patients.

**Results:**

*EGFR* amplification was detected in 18 of the 204 TNBC specimens (8.9 %) and was significantly associated with higher EGFR protein levels. Fourteen *PIK3CA* mutations were identified in exon 9 (6.7 %), and 17 in exon 20 (8.3 %). *PIK3CA* mutations, especially in exon 9, were significantly associated with grade I-II tumors. *PTEN* deletions were detected in 43 samples (21.50 %) and were significantly associated with grade III tumors (*p* < 0.001). Univariate analysis showed a significant association between relapse-free survival (RFS), T and N stage and exon 9 *PIK3CA* mutations. Overall survival was significantly associated with T stage, N stage and adjuvant chemotherapy, which was administered to 70.3 % of patients. In multivariate analyses, T stage, N stage, presence of exon 9 *PIK3CA* mutations and high EGFR protein level were independent poor prognostic factors for RFS, while adjuvant chemotherapy was associated with a better outcome.

**Conclusions:**

High EGFR protein expression and exon 9 *PIK3CA* activating mutations are independent prognostic factors in TNBC. The efficacy of anti-PI3K targeted therapies needs to be evaluated in this setting.

**Electronic supplementary material:**

The online version of this article (doi:10.1186/s12885-015-1977-3) contains supplementary material, which is available to authorized users.

## Background

Triple negative breast cancers (TNBC) occur most frequently in young women and tend to have a more aggressive behavior. They are characterized by a relapse rate that rapidly rises in the first 2 years after diagnosis, peaks at 2–3 years post-diagnosis and declines during the next 5 years [1]. Currently, chemotherapy is the only systemic therapeutic option for this tumor type because hormonal therapies and anti-HER2agents are ineffective due to the lack of expression of these therapeutic targets in tumor cells.

The transmembrane tyrosine kinase epidermal growth factor receptor (EGFR), which is encoded by the *EGFR* gene located on the short arm of chromosome 7, is frequently (30–52 %) overexpressed in TNBC [2], particularly in the basal-like subgroup, and is associated with poor prognosis [3]. EGFR activation through its tyrosine kinase domain leads to recruitment of downstream effectors and activation of proliferative and cell survival signaling pathways [4]. In historical reports, EGFR overexpression, using various detection methods, was observed in 14 to 91 % of breast tumors [5]. In more recent works, EGFR protein expression was detected in 16 to 36 % of breast cancers [6]. In addition, EGFR expression is part of the diagnostic criteria used to identify basal-like TNBC, a TNBC subgroup with worse prognosis [2]. However, the mechanisms responsible for EGFR expression in TNBC remain poorly understood. We previously reported [7], consistently with most of the published data [8, 9], the absence of *EGFR* activating mutations in TNBC samples from Caucasian patients. Therefore, the putative effect of EGFR TKIs in this population cannot be linked to activating mutations but, possibly, to EGFR overexpression or gene amplification [8–11]. Indeed, other *EGFR* modifications have been described in TNBC. Increased *EGFR* gene copy number has been inconstantly (0–51 %) reported in some EGFR-positive breast cancers [4, 8–12]. Cell membrane EGFR expression was associated with increased gene copy number in two of these studies [4, 12], but not with chromosome 7 polysomy in the report by Burness et al. [4]. Due to the high disparities in results and methods used for EGFR status evaluation, a comprehensive analysis of this putative target in TNBC is required.

The PI3K/PTEN pathway is involved both in EGFR downstream signaling and in TNBC physiopathology [13]. Mutations in *PIK3CA* (the gene encoding the p110 catalytic subunit of PI3K) and PTEN loss of expression (LOE) have been detected in breast cancers [14]. PTEN LOE has been observed in 50–82 % of basal-like breast cancers [15]. PTEN LOE appears to be the main cause of PI3K pathway alterations in breast cancer and is strongly associated with hormone receptor positivity [16], although it is observed also in 8–25 % of TNBC [11, 17, 18]. Conversely, *PIK3CA* mutations were detected in only a small fraction of TNBC with basal-like features in Martin's study [11]. The high frequency of PTEN LOE and the low occurrence of *PIK3CA* mutations in TNBC with basal-like features might bring support to the still debated hypothesis of the mutual exclusivity of these two alterations [14, 15].

Herein, we report the results of the analysis of *EGFR* gene amplification, EGFR expression and *PIK3CA* and *PTEN* deletion and their clinicopathological and prognostic implications in a large, comprehensive set of 204 European patients with TNBC.

## Methods

### Patients and tumor samples

A total of 1695 consecutive patients with breast cancer referred to the Val d’Aurelle Montpellier Cancer Institute (ICM) between 2002 and 2010 were prospectively entered in the database of a dedicated tumor DNA bank (Biobank number BB-0033-00059). Samples were isolated from frozen, histologically proven and macro-dissected invasive breast cancer specimens that were primarily handled for ER and PR testing by using the dextran charcoal method, as previously described [19, 20], or for uPA/PAI-1 quantification with the Femtelle® test. Tumors were considered as ER and PR positive when the receptor concentration was higher than 10 fmol/mg of protein (using the Dextran Charcoal Assay [DCC]), or > 10 % tumor cells were stained by immunohistochemistry (IHC) [21]. HER2 status was determined based on HER2 protein expression level by IHC using the A485 monoclonal antibody (Dako, Denmark). Tumors with HER2 scores of 0 and 1+ were considered as HER2 negative. In tumors with equivocal HER2 IHC test results (2+), gene amplification was evaluated using fluorescence or chromogenic (CISH) in situ hybridization. Specimens with HER2 3+ scores were considered as HER2 positive. Finally, 204 DNA samples from non-metastatic TNBC were selected for this study. Each individual treatment proposal was in accordance with our institution guidelines [22]. The clinicopathological characteristics and treatments of the 204 patients included in this study are summarized in Table 1. This study was reviewed and approved by the Montpellier Cancer Institute Institutional Review Board (ID number ICM-URC-2014/73). All patients gave their written, informed consent. As part of the study evaluated the prognostic impact of biological markers, this manuscript adheres to the REMARK guidelines.

**Table 1:** Patients and tumors’ characteristics

	Number of patients	(%)
	204	100
Age		
Median, range	56	29–86
<55 years	98	48.0
≥55 years	106	52.0
T		
T1	84	41.2
T2	97	47.5
T3	10	4.9
T4	13	6.4
N		
N0	132	64.7
N1	46	22.5
N2	18	8.8
N3	8	3.9
Histology		
Ductal	163	79.9
Lobular	13	6.4
Other^a^	28	13.7
SBR-EE grade		
1	9	4.5
2	60	29.9
3	132	65.7
NE	3	
Adjuvant chemotherapy		
No	60	29.7
Yes	142	70.3
Missing	2	

^a^6 invasive papillary carcinomas, 4 metaplastic carcinomas, 4 undifferentiated carcinomas, 3 apocrine carcinomas, 3 medullary carcinomas, 3 mixed ductal-lobular carcinomas, and one/each of the following histological subtypes: sarcomatoid carcinoma, adenoid cystic carcinoma, adenosquamous and neuroendocrine carcinoma

*NE* not evaluated

### Tissue processing and DNA extraction

DNA was extracted during tumor sample protein extraction protocol for either ER/PR or uPA PAI quantification. Briefly, each frozen tumor specimen was pulverized in liquid nitrogen and homogenized in a Polytron homogenizer (Glen Mills, NJ, USA) with a cytosol extraction buffer (20 mM Tris∙HCI, 1.5 mM ethylenediaminetetraacetic acid [EDTA], 10 mM Na2MoO4, 1.5 mM dithiothreitol and 10 % glycerol, pH 7.4; in the case of tumors processed for ER and PR testing using the DCC assay) or with Triton X-100 buffer (in the case of tumors processed for uPA/PAI-1 testing using the Femtelle kit, American Dignostica) with a buffer:tissue ratio of 10:1 (volume/weight) and centrifuged at 10,000 × g for 15 min. Total genomic DNA was extracted from the pellets obtained by centrifugation of either cytosol or Triton extract using the QIAamp DNA extraction minikit (ref 51304, Qiagen, Hilden, Germany) according to the manufacturer’s protocol. Supernatants were used to prepare cytosol or Triton X-100 protein extracts and the total protein content was quantified using the Pierce assay (BCA Protein Assay Kit, Pierce Biotechnology, Rockford, IL) as previously described [23].

### *PIK3CA* mutation detection

Polymerase Chain Reaction (PCR) amplification and High Resolution Melting [24] analysis were performed on a Rotor-Gene 6000™ instrument (Corbett Research, Mortlake, New South Wales, Australia) using the Light Cycler 480 HRM Master MIX kit (Ref 04 909 631 001, Roche Diagnostics, Meylan, France). Primers were designed to amplify *PIK3CA* fragments that span the exon 9 hotspot mutation region including the p.E542X and p.E545X mutations, and the exon 20 region including the hotspot mutations p.H1047X, p.H1048X and p.G1049X (Additional file 1: Table S1). Genomic DNA samples from MCF7 cells (c.1633G > A, p.E545K mutation) and T47D cells (c.3140 A > G, P.H1047R) were used as positive controls for *PIK3CA* exon 9 and exon 20 heterozygous mutations, respectively. Water (no template) was used to control for PCR contamination.

After HRM analysis, PCR products were purified using the ExoSAP-IT kit (ref US78200, GE Healthcare Life Sciences, Saclay, France) according to the manufacturer's instructions. Purified PCR products were then used as templates for sequencing with the Big Dye Terminator v1.1 kit (Ref 4336774, Applied Biosystems Inc., Foster City, CA). After migration completion, the *PIK3CA* sequences were analyzed with the Applied Biosystems Sequencing Analysis® software v5.2.

### *EGFR* amplification

Quantitative PCR (qPCR) used in this study was previously described [25]. Briefly, *EGFR* copy number was normalized to beta-actin (*ACTB*, 7p22.1), a gene located in the same chromosome, and to glyceraldehyde-3-phosphate dehydrogenase (*GAPDH*, 2p13.31), a gene located on another chromosome. We previously used this strategy [19] to distinguish between real copy number variations and aneusomy. One DNA sample with amplified *EGFR* was used as positive control (*EGFR/ACTB* ratio = 12.67 ± 2.31) and human placental DNA was used as normal control (*EGFR/ACTB* ratio = 0.55 ± 0.06). The theoretical threshold of gene amplification for a given sample was an *EGFR*/*ACTB* ratio = 2. Below this threshold, the sample was considered ‘wild type’ and above this threshold, the sample was considered ‘amplified’.

### Detection of *PTEN* and other chromosome 10 sequence copy number variations (CNV) by Multiplex Ligation-dependent Probe Amplification

Multiplex Ligation-Dependent Probe Amplification (SALSA MLPA probemix P225-D1 PTEN, MRC-Holland, Amsterdam, the Netherlands) is a high throughput, PCR-based method to determine the relative copy number of various human DNA target sequences. The method is based on the annealing of a mixture of oligonucleotides (Additional file 2: Table S2) to their cognate DNA sequences. DNA denaturation, hybridization, ligation, PCR and fragment analysis were performed according to the manufacturer’s specification. Raw data were visually controlled and then normalized using the Coffalyser.Net software. For each electropherogram, height peaks and areas under the peaks were exported to Coffalyser.NET. The algorithm is based on a double normalization (intra- and inter-sample) and calculates a quotient for each assay tube. The relative height of each individual probe peak, compared to the relative probe peak height of various reference DNA samples, reflects the relative copy number of the corresponding target sequence in the sample. Depending on the quotient value, DNA sequences were considered as normal, with heterozygous duplication, with heterozygous deletion, with homozygous deletion, or with non-interpretable results. This approach allows the identification of *PTEN* and chromosome 10q loss of heterozygosity (LOH) and aneuploidy.

### EGFR protein level

EGFR concentration in cytosol or Triton X-100 protein extracts was determined using the EGFR ELISA kit (MERCK ref CBA0*1*8), a sandwich enzyme immunoassay that employs specific goat anti-EGFR polyclonal antibodies. The range of standardization goes from 31.25 pg/ml to 2000 pg/ml. EGFR levels were standardized to the total protein content and results expressed in pg/mg of protein content. Because ranges of EGFR values were different according to sample preparation, EGFR expression was divided in terciles, for low, medium and high protein level.

### Statistical analysis

Categorical variables were presented as frequency distributions and continuous variables as medians and ranges. Categorical variables were compared with the Pearson’s chi-square or Fisher’s exact test. Differences were considered statistically significant at the *p* < 0.05 level. Overall survival (OS) was calculated from the date of surgery to the date of death (whatever the cause). Patients lost to follow-up were censored at the last documented visit. Relapse-free survival (RFS) was calculated from the surgery date to the recurrence date. Patients alive at the last follow-up without recurrence and patients lost to follow-up were censored at the time of the last follow-up. Patients who died without recurrence were censored at the date of death. The Kaplan-Meier method was used to estimate the OS and RFS rates. Differences in survival rates were compared using the log–rank test. Statistical analyses were performed with STATA 13.0 (StatCorp, College Station, TX).

## Results

### Patients and Tumor’s characteristics

For this study, 204 DNA samples from TNBC specimens that included a high percentage (>50 %) of tumor cells, as required for ER and PR or uPA/PAI-1 testing, were selected. The main clinicopathological characteristics of this cohort are summarized in Table 1 and were consistent with the classical TNBC features (i.e., a majority of T2+ tumors, one third of N+ cancers and high frequency of high grade tumors, as only nine tumor specimens [4.5 %, 3 ductal carcinomas, 3 ductal carcinomas with tubular inflexion, 1 lobular carcinoma, 1 invasive papillary carcinoma and 1 ductal carcinoma with cribriform inflexion] were classified as SBR-EE [Elston-Ellis modification of Scarff-Bloom-Richardson] grade 1). The patients’ median age was 56 years (range: 29–86 years). Ductal carcinoma was the most common histological type (79.9 %), 70.3 % of patients received adjuvant chemotherapy, while the remaining 29.7 % of patients received adjuvant radiation therapy if clinically indicated. None of the patients received additional hormonal therapy, targeted therapy or an investigational product.

### EGFR/PI3K/PTEN pathway alterations and clinicopathological correlations

**Fig. 1:**
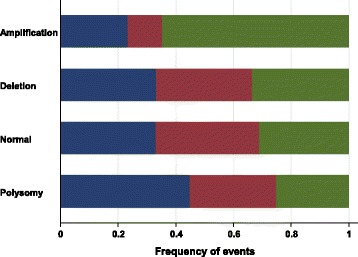
Correlation between EGFR gene status and EGFR protein levels (blue, lower tercile; red, middle tercile; green, upper tercile)

The results of the assessment of the EGFR/PI3K/PTEN axis alterations and of the correlations between *EGFR* amplification, EGFR protein level, *PIK3CA* mutation, *PTEN* status and clinicopathological characteristics are summarized in Table 2. As previously reported [7], no EGFR-activating mutation was identified in our population. *EGFR* amplification was detected in 18 tumor samples (8.9 %) and was significantly associated with higher EGFR protein levels compared to TNBC specimens with normal or deleted *EGFR* or chromosome 7 polysomy (*p* = 0.043, Fig. 1). *PIK3CA* mutations were identified in exon 9 (*n* = 14, 6.7 %) and in exon 20 (*n* = 17, 8.3 %) (Additional file 3: Table S3). The presence of *PIK3CA* mutations was significantly associated with SBR-EE grade I-II tumors (*p* = 0.038), particularly in the case of exon 9 mutations (64.3 % vs. 35.7 %, *p* = 0.02). *PTEN* deletions were detected in 43 TNBC specimens (21.5 %) and were significantly associated with SBR-EE grade III tumors (*p* < 0.001). No other statistically significant association was identified between EGFR/PI3K/PTEN pathway alterations and clinicopathological parameters.

**Table 2:** Correlations between *EGFR* amplification, EGFR protein level, *PIK3CA* mutation, *PTEN* status and clinicopathological characteristics

Patients’ characteristics	*EGFR* gene status									EGFR protein level							*PIK3CA* mutations							*PTEN* status				
	Normal		Deletion		Amplifi-cation		Polysomy		*p*	Lower tercile		Middle tercile		Upper tercile		*p*	Exon 9		Exon 20		No		*p*	Deletion		No deletion		*p*
	N	%	N	%	N	%	N	%		N	%	N	%	N	%		N	%	N	%	N	%		N	%	N	%	
Age								0.119							0.412							0.347					0.394	
<55 years	76	49.3	6	54.5	11	61.1	5	25.0		31	48.4	27	43.5	35	55.6		5	35.7	6	35.3	87	50.3		23	53.5	72	45.9	
≥55 years	78	50.7	5	45.5	7	38.9	15	75.0		33	51.6	35	56.5	28	44.4		9	64.3	11	64.7	86	49.7		20	46.5	85	54.1	
T status								0.878							0.836							0.384					0.387	
T1	63	40.9	4	36.4	9	50.0	8	40.0		25	39.1	25	40.3	28	44.4		8	57.1	8	47.1	68	39.3		15	34.9	67	42.7	
T2+	91	59.1	7	63.6	9	50.0	12	60.0		39	60.9	37	59.7	35	55.6		6	42.9	9	52.9	105	60.7		28	65.1	90	57.3	
N status								0.234							0.182							0.491					0.475	
N0	103	66.9	6	54.5	8	44.4	14	70.0		47	73.4	36	58.1	40	63.5		11	78.6	10	58.8	111	64.2		30	69.8	99	63.0	
N+	51	33.1	5	45.5	10	55.6	6	30.0		17	26.6	26	41.9	23	36.5		3	21.4	7	41.2	62	35.8		13	30.2	58	37.0	
SBR-EE grade								0.466							0.871							0.038					<0.001	
I-II	49	32.5	4	36.4	6	33.3	10	50.0		22	34.9	19	30.7	21	34.4		9	64.3	7	41.2	53	31.2		4	9.8	63	40.4	
III	102	67.5	7	63.6	12	66.7	10	50.0		41	65.1	43	69.3	40	65.6		5	35.7	10	58.8	117	68.8		37	90.2	93	59.6	
Histology								0.102							0.974							0.103					0.526	
Ductal	119	77.3	11	100	17	94.4	15	75.0		50	78.1	50	80.7	50	79.4		12	85.7	10	58.8	141	81.5		36	83.7	123	78.3	
Lobular and other	35	22.7	0	0.0	1	5.6	5	5.0		14	21.2	12	19.3	13	20.6		2	14.3	7	41.8	32	18.5		7	16.3	34	21.7	
*EGFR* Gene Status									-						0.164							0.873					0.224	
Normal	154	100	0	0.0	0	0.0	0	0.0		47	74.6	51	82.3	44	69.8		11	78.6	13	76.4	130	75.6		33	76.8	120	76.9	
Deletion	0	0.0	11	100	0	0.0	0	0.0		3	4.8	3	4.8	3	4.8		0	0.0	2	11.8	9	5.2		0	0.0	11	7.0	
Amplification	0	0.0	0	0.0	18	100	0	0.0		4	6.3	2	3.2	11	17.5		1	7.1	1	5.9	16	9.3		5	11.6	12	7.8	
Polysomy	0	0.0	0	0.0	0	0.0	20	100		9	14.3	6	9.7	5	7.9		2	14.3	1	5.9	17	9.9		5	11.6	13	8.3	
EGFR Protein level								0.164								-						0.180					0.110	
Lower tercile	47	33.1	3	33.3	4	23.5	9	45.0		64	100	0	0.0	0	0.0		3	27.3	4	26.7	57	35.0		9	21.4	53	36.8	
Middle tercile	51	35.9	3	33.3	2	11.8	6	30.0		0	0.0	62	100	0	0.0		7	63.6	4	26.7	51	31.3		14	33.3	47	32.6	
Upper tercile	44	31.0	3	33.3	11	64.7	5	25.0		0	0.0	0	0.0	63	100		1	9.1	7	46.6	55	33.7		19	45.3	44	30.6	
*PIK3CA* Mutations								0.873							0.180								-				0.565	
Exon 9	11	7.1	0	0.0	1	5.6	2	10.0		3	4.7	7	11.3	1	1.60		14	100	0	0.0	0	0.0		1	2.3	12	7.6	
Exon 20	13	8.4	2	18.2	1	5.6	1	5.0		4	6.2	4	6.4	7	11.1		0	0.0	17	100	0	0.0		3	7.0	12	7.6	
No	130	84.5	9	81.8	16	88.8	17	85.0		57	89.1	51	82.3	55	87.3		0	0.0	0	0.0	173	100		39	90.7	133	84.8	
*PTEN* Status								0.224							0.110							0.565						-
Deletion	33	21.6	0	0.0	5	29.4	5	27.8		9	14.5	14	23.0	19	30.2		1	7.7	3	20.0	39	22.7		43	100	0	0.0	
No deletion	120	78.4	11	100	12	70.6	13	72.2		53	85.5	47	77.0	44	69.8		12	92.3	12	80.0	133	77.3		0	0.0	157	100

### Survival analyses

**Fig. 2:**
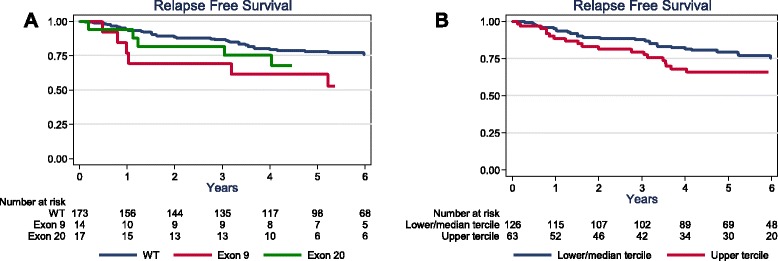
Relapse-Free Survival rates in patients with TNBC (*n* = 204) in function of time according to **a** *PIK3CA* mutations, **b** EGFR protein levels (terciles)

Using May 1, 2014 as cut-off date, the median follow-up was 6.4 years (range: 0.1–12.8 years). Forty seven deaths (5-year OS: 81.3 % [74.8–86.3]) and 52 relapses occurred (5-year RFS: 76.1 % [69.2–81.6]). The relapse pattern of our population was consistent with the previously reported relapse risk temporal distribution [1, 26], as most relapses occurred during the first 3 years of follow-up (Additional file 4: Figure S1 and Additional file 5: Figure S2). Univariate analysis (Table 3) showed a significant association between RFS and T stage, N stage and exon 9 *PIK3CA* mutations and a marginal association (*p* = 0.07) with adjuvant chemotherapy. OS was significantly associated with T stage, N stage, adjuvant chemotherapy and marginally with exon 9 *PIK3CA* mutations (*p* = 0.063) and *EGFR* status (Polysomy/Amplification vs. Deletion/Normal, *p* = 0.075). As some patients died from non-TNBC related causes, a multivariate analysis was performed using RFS data to identify independent, TNBC-specific prognostic factors. T stage, N stage, presence of exon 9 *PIK3CA* mutations and high EGFR protein level were identified as independent poor prognostic factors, while the use of adjuvant chemotherapy was statistically associated with a better outcome (Table 4, Fig. 2). These parameters remained significant independent prognostic factors in the two subgroup analyses (in the population of tumors excluding the 11 cases with HR values between 1 % and 9 % [*n* = 193], as well as in the ductal tumors population [*n* = 163]).

**Table 3:** Univariate analysis

	N	RFS			OS		
		Events	5-y RFS	HR (95 % CI)	Events	5-y OS	HR (95 % CI)
Age							
<55 years	98	21	79.9	1	17	84.1	1
≥55 years	106	31	72.6	1.29 [0.74; 2.24]	30	78.9	1.51 [0.83; 2.73]
		*p* = 0.370			*p* = 0.174		
T							
T1	84	11	88.3	1	11	90.7	1
T2+	120	41	67.5	2.91 [1.50; 5.67]	36	74.7	2.48 [1.26; 4.88]
		*p* = 0.001			*p* = 0.006		
N							
N-	132	20	86.9	1	21	89.0	1
N+	72	32	56.1	3.56 [2.03; 6.22]	26	66.8	2.58 [1.45; 4.59]
		*p* < 0.001			*p* = 0.001		
SBR-EE grade							
1-2	69	17	79.9	1	15	86.3	1
3	132	35	73.7	1.13 [0.63; 2.02]	32	78.4	1.21 [0.65; 2.24]
		*p* = 0.676			*p* = 0.541		
Histology							
Ductal	163	42	75.4	1	40	80.0	1
Lobular/Other	41	10	78.7	0.93 [0.47; 1.86]	7	86.6	0.67 [0.30; 1.49]
		*p* = 0.848			*p* = 0.325		
Adjuvant CT							
No	60	21	66.7	1	24	69.1	1
Yes	142	31	80.1	0.60 [0.35; 1.05]	23	86.8	0.40 [0.23; 0.71]
		*p* = 0.070			*p* = 0.001		
*EGFR* status							
Normal/Deletion	165	41	78.0	1	35	81.8	1
Ampl./Polysomy	38	11	65.9	1.36 [0.69; 2.65]	12	78.5	1.80 [0.93; 3.49]
		*p* = 0.368			*p* = 0.075		
*PIK3CA* status							
No mutation	173	40	78.0	1	36	83.2	1
Exon 9	14	7	61.5	2.62 [1.17; 5.86]	7	69.2	2.55 [1.13; 5.74]
Exon 20	17	5	67.8	1.38 [0.54; 3.50]	4	70.5	1.23 [0.44; 3.46]
		*p* = 0.048			*p* = 0.063		
EGFR protein level							
Lower and middle tercile	126	30	79.4	1	27	84.1	1
Upper tercile	63	20	66.0	1.54 [0.87; 2.72]	17	73.8	1.42 [0.78; 2.62]
		*p* = 0.131			*p* = 0.247		
*PTEN* status							
No deletion	157	38	78.5	1	33	83.4	1
Deletion	43	13	67.7	1.38 [0.74; 2.60]	13	75.1	1.66 [0.87; 3.16]
		*p* = 0.309			*p* = 0.117

**Table 4:** Multivariate analysis (RFS)

	HR	95 % CI	*p*
T			0.008
T1	1		
T2+	2.48	[1.21; 5.07]	
N			<0.001
N-	1		
N+	4.17	[2.23; 7.78]	
Adjuvant CT			0.003
No	1		
Yes	0.39	[0.22; 0.70]	
*PIK3CA* Exon 9 Mutation			0.001
No	1		
Yes	6.38	[2.42; 16.8]	
EGFR protein level			0.011
Lower and middle tercile	1		
Upper tercile	2.22	[1.22; 4.03]

## Discussion

We performed an extensive analysis of the EGFR/PI3K/PTEN axis alterations and of their clinicopathological and prognostic implications in a set of 204 European patients with TNBC. The results obtained in our series are in accordance with comprehensive evaluations of the mutational spectrum of TNBC reported in the literature [27]. We previously reported [7] the absence of EGFR-activating mutation in this series of TNBC. We showed a statistically significant association between *EGFR* gene amplification and EGFR protein expression. As we did not chose to classify our series of tumors as basal-like or non-basal-like, but rather to evaluate quantitatively the level of EGFR protein expression, it appeared that a quantitative continuous test such as the one offered by the biochemical assay was the more relevant to achieve our objective, and thus selected this method for the EGFR protein evaluation. Our results are consistent with the study by Bhargava et al. [8] who found a correlation between *EGFR* gene amplification (detected in 6 % of their breast cancer patients) and EGFR overexpression. Interestingly, 73 % of tumors harboring *EGFR* gene amplification were TNBC (the total number of TNBC in this series was not reported). However, they could not differentiate between chromosome 7 polysomy and true *EGFR* gene amplification, as they used only one *EGFR* gene probe for CISH. Two recent reports [11, 28] also found a significant correlation between EGFR protein overexpression (evaluated by IHC) and *EGFR* gene amplification and chromosome 7 polysomy by dual probe (*EGFR*/CEP7) FISH analysis. However, as concomitant evaluation of the prognostic value of *EGFR* gene alterations and EGFR protein expression (by IHC and biochemically) has never been reported, it is currently impossible to say which is the best method(s) to assess EGFR status as a prognostic indicator in TNBC.

The PI3K pathway is up-regulated in basal-like cancers and in TNBC. The majority of the reported mutations is located within the helical (exon 9) and the kinase (exon 20) domains of the PI3K catalytic subunit p110 alpha and result in constitutive PI3K activation, AKT signaling induction and oncogenic transformation [29]. In our TNBC cohort, exon 9 *PIK3CA* activating mutations were detected in 6.7 % and exon 20 mutations in 8.3 % of tumor samples, in agreement with previously published reports on *PIK3CA* mutations in patients with TNBC [14, 30–32]. The frequency and clinical impact of *PIK3CA* mutations need to be assessed in homogeneous biological subgroups of breast cancers, as the prevalence and biological implications of these mutations appear to vary in the different subgroups [13, 14, 31]. Arsenic et al. reported an overall *PIK3CA* mutation frequency of 15.8 % in breast cancer and of 13.2 % in TNBC [30]. Moreover, mutations in exon 20 were more numerous compared with those in exon 9, in agreement with other works [14, 31] and our results. Although exon 9 and exon 20 *PIK3CA* mutations do not appear to be mutually exclusive and they could have a synergistic effect [33], they were never concomitantly detected in our cohort.

Then we showed that exon 9 *PIK3CA* mutations were independent prognostic factors in TNBC while exon 20 *PIK3CA* mutations did not. Previous studies analyzed *PIK3CA* mutation prognostic impact in the whole breast cancer population and not specifically in TNBC [34], or evaluated together exon 9 and exon 20 *PIK3CA* mutations [32, 35], or analyzed their prognostic impact in small series (<100 patients with TNBC) [30, 35]. Interestingly, Zhao et al. [33] showed that *PIK3CA* mutations in exon 9 and exon 20 could be associated with different activation mechanisms. Specifically, the gain of function induced by exon 9 *PIK3CA* mutations is independent of binding to the PI3K regulatory subunit p85, but requires interaction with RAS-GTP. Conversely, exon 20 mutants do not require RAS-GTP binding, but are highly dependent on the interaction with p85. These molecular differences could, at least partially, explain the differential prognostic impact of exon 9 and 20 *PIK3CA* mutations in our series.

TNBC activation of downstream members of the PI3K signaling pathway is very common as indicated by the frequency of PTEN LOE, *INPP4B* loss and *MAGI3* and *AKT3* activating translocations [36]. As we wanted to evaluate the most stable PTEN modifications, we considered that genomic alterations were the most relevant way to identify stable PTEN loss of function. Thus, the evaluation of *PTEN* deletions using the MLPA methods appeared the most robust way to identify a true PTEN deficiency in our population. *PTEN* deletions were detected in 21.50 % and were significantly associated with grade III tumors (*p* < 0.001). *PIK3CA* mutations and PTEN loss are considered to be nearly mutually exclusive in breast tumors [11, 14], a characteristic shared by our series, as only four tumors presented concomitantly a *PTEN* deletion and a *PIK3CA* activating mutation (one in exon 9, three in exon 20, Table 2). PTEN dysregulation, observed in 16.6 to 63 % of TNBC depending on the evaluation method and the disease stage [17, 35, 37, 38], has been consistently reported to be associated with poor prognosis in these patients [37, 38]. The lack of significant association between *PTEN* deletion and RFS/OS in our study could be linked to the fact that we evaluated exclusively *PTEN* deletions, rather than PTEN LOE, and the frequency of PTEN abnormalities (20.1 %) was thus lower than in classical LOE studies [17, 35, 37, 38]. An extensive evaluation of the different mechanisms responsible for PTEN LOE, together with PTEN expression by IHC, could be useful for understanding its clinical and biological implications in our population.

A potential pitfall of our study could be linked to the choice of a European definition of TNBC, considering a <10 % negativity threshold for the determination of the hormone receptor status. However, the fact that the exclusion of the 11 cases with HR values between 1 and 9 % did not change the multivariate analysis results, and the fact that recent biological studies showed a close biologic similarity between the <1 % HR and 1–9 % HR population [39], both strengthen our results.

## Conclusions

High EGFR protein expression and exon 9 *PIK3CA* activating mutations are independent prognostic factors in TNBC. These different molecular abnormalities could affect TNBC sensitivity to various anticancer treatments under development, such as mTOR inhibitors, PARP inhibitors or androgen receptor inhibitors. Therefore, it is now important to globally evaluate the EGFR/PI3K/PTEN pathway, together with androgen receptor and DNA repair deficiency status, to better identify different TNBC subgroups that could be sensitive to various kinase inhibitors, including anti-PI3K targeted therapies, or specific targeted therapy combinations.

## Additional files

Additional file 1: Table S1. Primers for *PIK3CA* exon 9 and 20 mutation analysis. (DOCX 17 kb) Additional file 2: Table S2. Mix of probes in the Salsa MPLA kit for the detection of PTEN and Chr10 genomic abnormalities EX: exon; LPO: 5' half of the probe, RPO: 3' half of the probe. (DOCX 44 kb) Additional file 3: Table S3. Exon 9 and 20 PIK3CA mutations in triple negative breast cancer specimens. (DOCX 20 kb) Additional file 4:
**Relapse Free and Overall Survival.** (PPTX 55 kb) Additional file 5:
**Relapse Rates of the patients with TNBC according to time (** ***n*** **=204).** (PPTX 103 kb)
