# Supplementary material for: High EGFR protein expression and exon 9 PIK3CA mutations are independent prognostic factors in triple negative breast cancers
Source: BMC Cancer. 2015 Dec 18;15:986. doi: 10.1186/s12885-015-1977-3 (PMC4683760; doi:10.1186/s12885-015-1977-3)
Supplement: Additional file 1: Table S1. — Primers for PIK3CA exon 9 and 20 mutation analysis. (DOCX 17 kb) [file 12885_2015_1977_MOESM1_ESM.docx]

Supplementary Table 1: Primers for *PIK3CA* exon 9 and 20 mutation analysis

| Amplicon | Primers | Tm | %GC |
| --- | --- | --- | --- |
| Exon 9 forward | 5’GCTAGAGACAATGAATTAAGGGAAA3’ | 58.1 | 36 |
| Exon 9 reverse | 5’CTCCATTTTAGCACTTACCTGTGAC3’ | 61.3 | 44 |
| Exon 20 forward | 5’AAACTGAGCAAGAGGCTTTGGA3’ | 58.4 | 45.5 |
| Exon 20 reverse | 5’TGGAATCCAGAGTGAGCTTTCA3’ | 58.4 | 45.5 |
