# Supplementary material for: High EGFR protein expression and exon 9 PIK3CA mutations are independent prognostic factors in triple negative breast cancers
Source: BMC Cancer. 2015 Dec 18;15:986. doi: 10.1186/s12885-015-1977-3 (PMC4683760; doi:10.1186/s12885-015-1977-3)
Supplement: Additional file 2: Table S2. — Mix of probes in the Salsa MPLA kit for the detection of PTEN and Chr10 genomic abnormalities EX: exon; LPO: 5' half of the probe, RPO: 3' half of the probe. (DOCX 44 kb) [file 12885_2015_1977_MOESM2_ESM.docx]

| Probe Number | Gene | Ex | chr. pos. | LPO | RPO | start | end | Length  (bp) |
| --- | --- | --- | --- | --- | --- | --- | --- | --- |
| S0750-L21493 | SLC9A2 | 2 | 02q12.1 | GTATGCTGTGGTAGGGACACTTTGGA | ATTCCATTGGCATTGGGGTGTCTTTGTTAAGTGCCTGGCCAGTAGCGCT  GTCCTGT | 102640674 | 102640731 | 125 |
| 00797-L25925 | IL4 | 1 | 05q31.1 | CTCACATTGTCACTGCAAATCGACACCTAT | TAATGGGTCTCACCTCCCAACTGCTTCCCCCT | 132037613 | 132037671 | 131 |
| 16316-L25926 | RAB7A | 3 | 03q21.3 | TCAGAAATTCAGCAATCAGTACAAAGCCACAATAGGAGC | TGACTTTCTGACCAAGGAGGTGATGGTGGATG | 129999512 | 129999582 | 136 |
| 10345-L25028 | KLLN | 1 | 10q23.31 | GTCTGAGAGCTTTCATTTTTAGGGCAAACGAGCCGAGT | TACCGGGGAAGCGAGAGGTGGGGCGCTGCAAG | 89612478 | 89612547 | 142 |
| 07632-L22241 | GATA3 | 1 | 10p14 | TGAGCTTCACTGAGGGAGAGCGAGACAGAGCGAGCAACGCAAT | CTGACCGAGCAGGTCGTACGCCGCCGCCTCCTCCT | 8136771 | 8136837 | 148 |
| 13690-L15159 | PTEN | 6 | 10q23.31 | CCAGTCAGAGGCGCTATGTGTATTATTATAGCTACCTGT | TAAAGAATCATCTGGATTATAGACCAGTGGCACTGTTGTTTCA | 89701868 | 89701949 | 154 |
| 07685-L23140 | PTEN | 8 | 10q23.31 | CATTTTGTGGGGGTTGTTGACTTGTATGTA | TGTGATGTGTGTTTAATTCTAGGAGTACAGCTGATGAAGAACTTGC | 89710865 | 89710946 | 160 |
| 14281-L15951 | OCA2 | 13 | 15q13.1 | TCCAAGAAGGCAGAGAGGACGGCCGCGATGAGA | CAGAGCATGATGATCATGGCCCACACCCGTCCCC | 25903847 | 25903913 | 166 |
| 17388-L08261 | KLLN | 1 | 10q23.31 | CACCGGAGCGGGCGCAGGAGA | GGCCTGCGGGGTGCGTCCCACTCACAGGGAT | 89612348 | 89612401 | 172 |
| 17314-L20922 | PTEN | 3 | 10q23.31 | GACATTGTTGGCACAGATTCATGTTACTTGATCTGCTTT | AAATGACTTGGCATCTAGCCCATATTTGAGCCCATAACCGTGTGGT | 89675483 | 89675566 | 178 |
| 13686-L15155 | KLLN | 1 | 10q23.31 | TTCCAGGCAGCTACACTGGGCATGCTCAGTAGA | GCCTGCGGCTTGGGGACTCTGCGCTCGCACCCAGAGCTAC | 89613066 | 89613136 | 183 |
| 06729-L06339 | PTEN | 2 | 10q23.31 | TGGAGTCCAGGAAATGATATCACATAAGT | ACCTGATTATGTAATGTATAAGGTCAGTTCTTATCAAATGAACTGTATCC | 89644014 | 89644099 | 190 |
| 11393-L12119 | USH2A | 23 | 01q41 | GGAAAATGGCATGAAATAATTGCTATTAGGCA | TCAGGCTTTTGGCCAAATCACTCTGGATGGGATATATACAGGTA | 214328975 | 214329050 | 196 |
| 18254-L22971 | PTEN | 1 | 10q23.31 | TGCTCTACTCTCAAACTTCCATCATGGCTGCAGCTTCC | GAGAGGAGAGAACTGAGCGCAGTCGCGTCCC | 89613344 | 89613403 | 202 |
| 17391-L21278 | PTEN | 4 | 10q23.31 | CCTCGGTGTCATAATGTCTTTCAGCACAACTAAAAGAAAAGTT | TAAAAGTGATATAAAGTTATCTTTTGCACTTAGGAAGAAAAAAAAGTACTA  ATACT | 89680713 | 89680805 | 208 |
| 07686-L15591 | PTEN | 9 | 10q23.31 | GTAAGTTGTTGACTGATGTAGGTACTAACAGCATCTGAA | TTTTAGCACTGGCCTTGATTACACAGGAGATGGAGAAGTCG | 89716113 | 89716189 | 214 |
| 08219-L24929 | FGFR2 | 21 | 10q26.13 | GTGTAATCTGCATTCATCTTGCACGGCTAT | TGCAAAGTGAGTGGGTGTTTCCAAAGCAAAACACAATACTTTAGTACAGAA | 123227968 | 123228048 | 220 |
| 17387-L26030 | PTEN | 1 | 10q23.31 | GCAGCTACCGTCAAGTCCAGAGCCATTTCCATCCTGCAGAAGAA | GCCCCGCCACCAGCAGCTTCTGCCATCTCTCTCCTCCTTTTTCTTCAGC | 89614100 | 89614191 | 227 |
| 10267-L26029 | LCA5 | 2 | 06q14.1 | GGTTTATGATAGACGTTGTTTTTAGCCCAGGTGTAAAAGA | TGGGTCCTAGGAATTGGTCCAGCTGGTCTGCTAAAAC | 80291764 | 80291838 | 232 |
| 08751-L26028 | PCDH15 | 5 | 10q21.1 | GGTGCAGGTACAATTCTGGTGGACAACATGCTGA | TCAAAGGGACTGCTGGAGGACCAGACCCCACCATAGAACT | 55808642 | 55808713 | 238 |
| 10384-L26027 | COL5A1 | 19 | 09q34.3 | GTGACCTCCGGGAGACGATGGAGAA | AGGGTAGGTATTCTGCCGTCCCTCCGACTGC | 136793620 | 136793673 | 245 |
| 17393-L22030 | CELF2 | 4 | 10p14 | GAAGATGTTTGTCGGACAGATCCCCCGGTCAT | GGTCGGAAAAGGAGCTGAAAGAACTTTTTGAGCCTTACGGAGCC | 11247522 | 11247596 | 252 |
| 07192-L16235 | BMPR1A | 4 | 10q23.2 | TAACTCTTAAGAAGGGCTGCATAAAATACTTACATGCA | TGTGTTATTAATAGCATCATCTGGACAGTGCCCT | 88639922 | 88639990 | 261 |
| 08938-L22242 | SLC6A5 | 7 | 11p15.1 | GAGTACTTTGTGCTGAAGATTTCTGCAGGGA | TTGAATATCCTGGCGAGATCAGGTGGCCACTAGCTCTCTGCCTC | 20595874 | 20595946 | 267 |
| 19294-L25714 | LGI1 | 6 | 10q23.33 | CTGGGTAATTCATTTAATTGTGACTGTAAACTGAAATGGCTA | GTGGAATGGCTTGGCCACACCAATGCAACTGTTG | 95542499 | 95542572 | 274 |
| 17308-L25183 | PTENP1 | 1 | 09p13.3 | GCTAAAGAGCTTTGTGATATACTGGTTCACATCA | TACCCCTTTGCACTTGTGGCAACAGATAAGTTTGCAGTTAGCTA | 33664992 | 33665069 | 280 |
| 17390-L14811 | PTEN | 2 | 10q23.31 | CAGTTTGATTGCTGCATATTTCAGATATTTCTTTCCTTAACTAAAGTACTCAG | ATATTTATCCAAACATTATTGCTATGGGATTTCCTGCAGAAAGACTTG | 89643712 | 89643809 | 285 |
| 18081-L23071 | RET | 2 | 10q11.21 | GACGAGGACACCGGCCTCCTCTACCTTA | ACCGGAGCCTGGACCATAGCTCCTGGGAGAAGCTCAGTGTCCGCAGT | 42916107 | 42916178 | 292 |
| 04570-L25184 | SLC12A3 | 23 | 16q13 | CACTCCAGGATCATTTCTCTGCTGAGCAAGTTCCGACT | GGGATTCCATGAAGTCCACATCCTCCCTGACATCAACCAG | 55490937 | 55491012 | 299 |
| 18380-L25185 | ANXA7 | 4 | 10q22.2 | GTCCAGGCCAAGGATTTGGAGTCCCACCAGG | TGGAGCAGGCTTTTCTGGGTATCCACAGCCACCT | 74826971 | 74827034 | 305 |
| 17305-L21055 | ESCO2 | 1 | 08p21.1 | CCAGCGGCTTCCTCCTAGCCTGGCGCGCGATT | ATTTGAAGACGCTCACGGAGCGGCTGGCTAGGCTGA | 27687955 | 27688015 | 312 |
| 03639-L21321 | PTEN | 6 | 10q23.31 | CGAAGGATGAGAATTTCAAGCACTTACTGCAAGT | TCCGCCACTGAACATTGGAATAGTTTCAAACATCATCTTGTGAAAC | 89701944 | 89702026 | 319 |
| 19293-L25664 | PTEN | 3 | 10q23.31 | CCTTAAACCATTACAAGATATACAATCTGTAAGTATGTTTTCTTATTTGTATGCTT | GCAAATATCTTCTAAAACAACTATTAAGTGAAAGTTATCTGCTTGTTAGAGTGAGG | 89675271 | 89675378 | 328 |
| 17396-L25927 | PTEN | 9 | 10q23.31 | GTGGATGTATAGGGTAAAACAAGATTGGTCAGGAAAAGAGAATTGTTC | CTATAACTGGTAATCTGACACAATGTCCTATTGCCATTA | 89715284 | 89715373 | 337 |
| 18694-L24032 | PTEN | 3 | 10q23.31 | CATGATTACTACTCTAAACCCATAGAAGGGGTATTTGTT | GGATTATTTATTTTCACTTAAATGGTATTTGAGATTAGGAAAAAGAAAATCTGTCTTTTGGT | 89675056 | 89675166 | 344 |
| 05273-L25208 | SPAST | 10 | 02p22.3 | CTTATAGAAGGTTGAAGTTCTCGAGCCACAGCA | AAAAGAGCCCTCACCAATTTCTCTCCTTCTCCCACCT | 32215134 | 32215202 | 352 |
| 17397-L25715 | PTEN | 8 | 10q23.31 | GCATTTGCAGTATAGAGCGTGCAGATAATGACAAGGAA | TATCTAGTACTTACTTTAACAAAAAATGATCTTGACAAAGCAAATAAAGACAAAGCCAACCGATA | 89710734 | 89710836 | 359 |
| 18110-L25928 | KLLN | 1 | 10q23.31 | CCTACCCAGCCAGGCTCCGCGCGCCCCGGCCGGA | CCGTGCACGTTTGGGGTTACCGGGTTGAGTGGAAAGTA | 89612147 | 89612210 | 364 |
| 08602-L25186 | HTRA1 | 2 | 10q26.13 | CGATACCTGCCGTGGTTCATATCGAATTGTTTCGCA | AGTAAAGAGAGCCTTCCTTTTTCCTATAACCTCCGAAGCTTTCACCG | 124238476 | 124238553 | 373 |
| 03638-L24933 | PTEN | 5 | 10q23.31 | CCATTCACTGTAAAGCTGGAAAGGGACGAACTGGTGTAATGATA | TGTGCATATTTATTACATCGGGGCAAATTTTTAAAGGCACAAGAGGC | 89682864 | 89682948 | 379 |
| 13248-L24934 | COL11A1 | 31 | 01p21.1 | CTGTCAAATTGGACCAAGAGGGGAAGATGGCCCTGAAGGA | CCCAAAGGTCGAGCAGGCCCAACTGGAGACCC | 103225805 | 103225869 | 388 |
| 17825-SP0555-L22726 | PTENP1 | 1 | 09p13.3 | CCTGGCCCGGGCTCCGGAGGCCGCTGGA | GTCTTCTCCCCATTCCGCTGCCGCCGCTGCCAGGCCTC | 33666861 | 33666952 | 399 |
| 13032-L22244 | PTEN | 5 | 10q23.31 | TCTATGGGGAAGTAAGGACCAGAGACAAAAAGGTAAGTT | ATTTTTTGATGTTTTTCCTTTCCTCTTCCTGGATCTGAGAATTTATTGGAAA | 89682957 | 89683047 | 409 |
| 09793-L25209 | SPG11 | 32 | 15q21.1 | CCATCAGGCCTTCATCAGCACACAGGGCCTTA | AGCCAGATACTGTGGCTGAACTCGTGGCAGAAGAGGTG | 42653071 | 42653136 | 418 |
| 08839-L22026 | DYSF | 58 | 02p13.2 | CCTTCTCCCTCCAGAACTATGCTGCCATGAAGCT | GGTGAAGCCCTTCAGCTGAGGACTCTCCTGCCCTGTAG | 71767082 | 71767149 | 427 |
| 13692-L21061 | PTEN | 7 | 10q23.31 | CCTCTTCCACAAACAGAACAAGATGCTAAAAAAGGTTT | GTACTTTACTTTCATTGGGAGAAATATCCAAAATAAGGACAGATTAAAAG | 89707721 | 89707810 | 436 |
| 17395-L21062 | PTEN | 9 | 10q23.31 | CCGATGAGTCATATTTGTGGGTTTTCATTTTAAATTTTCTTT | CTCTAGGTGAAGCTGTACTTCACAAAAACAGTAGAGGAGCCGTCA | 89714978 | 89715062 | 445 |
| 17392-L21057 | ITIH5 | 15 | 10p14 | CCTTATCTCGAGATCACACCGAGCAGAGTCA | TCTTGGATGGTGGGGACAGACTGGTGCTCCCCTGCAACCAGAG | 7648193 | 7648265 | 454 |
| 17394-L21385 | PTEN | 1 | 10q23.31 | CCAAGAGGATGGATTCGACTTAGACTTGACCTGTATC | CATTTCTGCGGCTGCTCCTCTTTACCTTTCTGTCACTCTCTTAGAA | 89614254 | 89614336 | 465 |
| 17386-L22174 | PTEN | 7 | 10q23.31 | CCATATTCCTCCAATTCAGGACCCACACGACGGGAA | GACAAGTTCATGTACTTTGAGTTCCCTCAGCCGTTACCTG | 89707630 | 89707700 | 475 |
| 13594-L22376 | CACNA1A | 35 | 19p13.2 | CCAGGTTGAGCTGGGTTTCCCAGAAGCCACTGGAGGAATG | GCAGCCCCTGGTCGTCACCCTCCAATTCCACAGGTGT | 13206847 | 13206921 | 485 |
| 17389-L21835 | PTEN | 4 | 10q23.31 | CCTCACTCGATAATCTGGATGACTCATTATTGTTATGA | CAGTAAGATACAGTCTATCGGGTTTAAGTTATACAACATAGTACAGTACATTCATACCTACC | 89680826 | 89680927 | 493 |
| 15203-L20113 | GBE1 | 7 | 03p12.2 | CCAGATTCCTGTTATTTTCATTCTGGACCTAGAGGGA | CTCATGATCTTTGGGATAGCAGATTGTTTGCCTACTCCAGGTAGGTACA | 81774613 | 81774698 | 500 |

Supplementary Table 2: Mix of probes in the Salsa MPLA kit for the detection of PTEN and Chr10 genomic abnormalities

EX: exon; LPO: 5' half of the probe, RPO: 3' half of the probe.
