# Supplementary material for: High EGFR protein expression and exon 9 PIK3CA mutations are independent prognostic factors in triple negative breast cancers
Source: BMC Cancer. 2015 Dec 18;15:986. doi: 10.1186/s12885-015-1977-3 (PMC4683760; doi:10.1186/s12885-015-1977-3)
Supplement: Additional file 3: Table S3. — Exon 9 and 20 PIK3CA mutations in triple negative breast cancer specimens. (DOCX 20 kb) [file 12885_2015_1977_MOESM3_ESM.docx]

Supplementary Table 3. Exon 9 and 20 PIK3CA mutations in triple negative breast cancer specimens

|  | | **N=204** | **%** |
| --- | --- | --- | --- |
| **Mutation Exon 9** | |  |  |
|  | No | 190 | 93.1 |
|  | Yes | 14 | 6.9 |
| **Mutation Exon 9 (N=14)** | |  |  |
|  | c.1624 G>A, p.E542K | 3 | 21.4 |
|  | c.1633 G>A, p.E545K | 8 | 57.2 |
|  | c.1634 A>C, p.E545A | 1 | 7.1 |
|  | c.1634 A>G, p.E545G | 2 | 14.3 |
| **Mutation Exon 20** | |  |  |
|  | No | 187 | 91.7 |
|  | Yes | 17 | 8.3 |
| **Mutation Exon 20 (N=17)** | |  |  |
|  | c.3140 A>G, p.H1047R | 10 | 58.8 |
|  | c.3140 A>T, p.H1047L | 5 | 29.4 |
|  | c.3145 G>C, p.G1049R | 1 | 5.9 |
|  | c.3145 G>T, p.G1049C | 1 | 5.9 |
