# Supplementary figures and images for: High EGFR protein expression and exon 9 PIK3CA mutations are independent prognostic factors in triple negative breast cancers
Source: BMC Cancer. 2015 Dec 18;15:986. doi: 10.1186/s12885-015-1977-3 (PMC4683760; doi:10.1186/s12885-015-1977-3)

## Slide 1
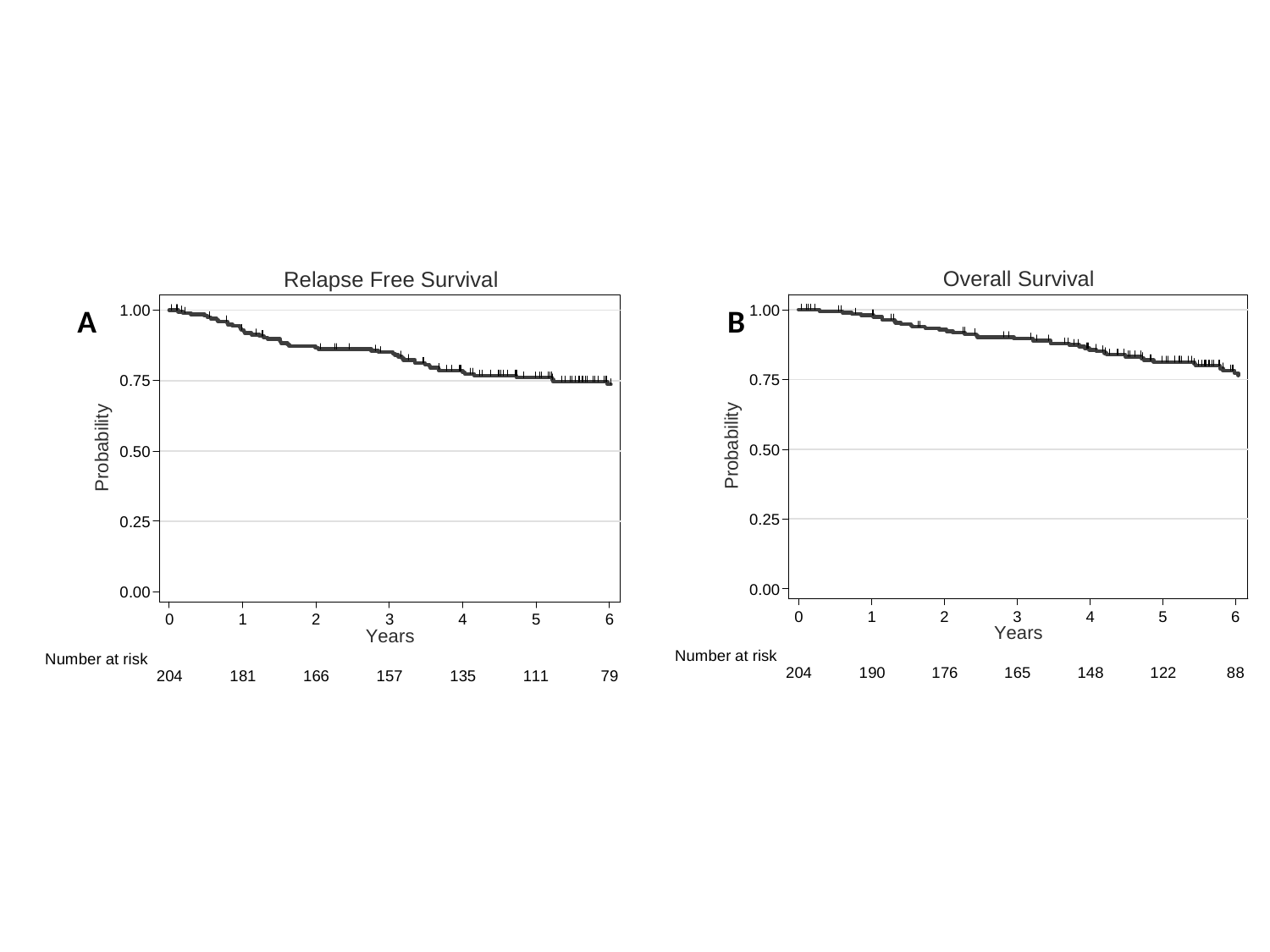

A
B

Supplement: Additional file 4: — Relapse Free and Overall Survival. (PPTX 55 kb) [file 12885_2015_1977_MOESM4_ESM.pptx]

## Slide 1
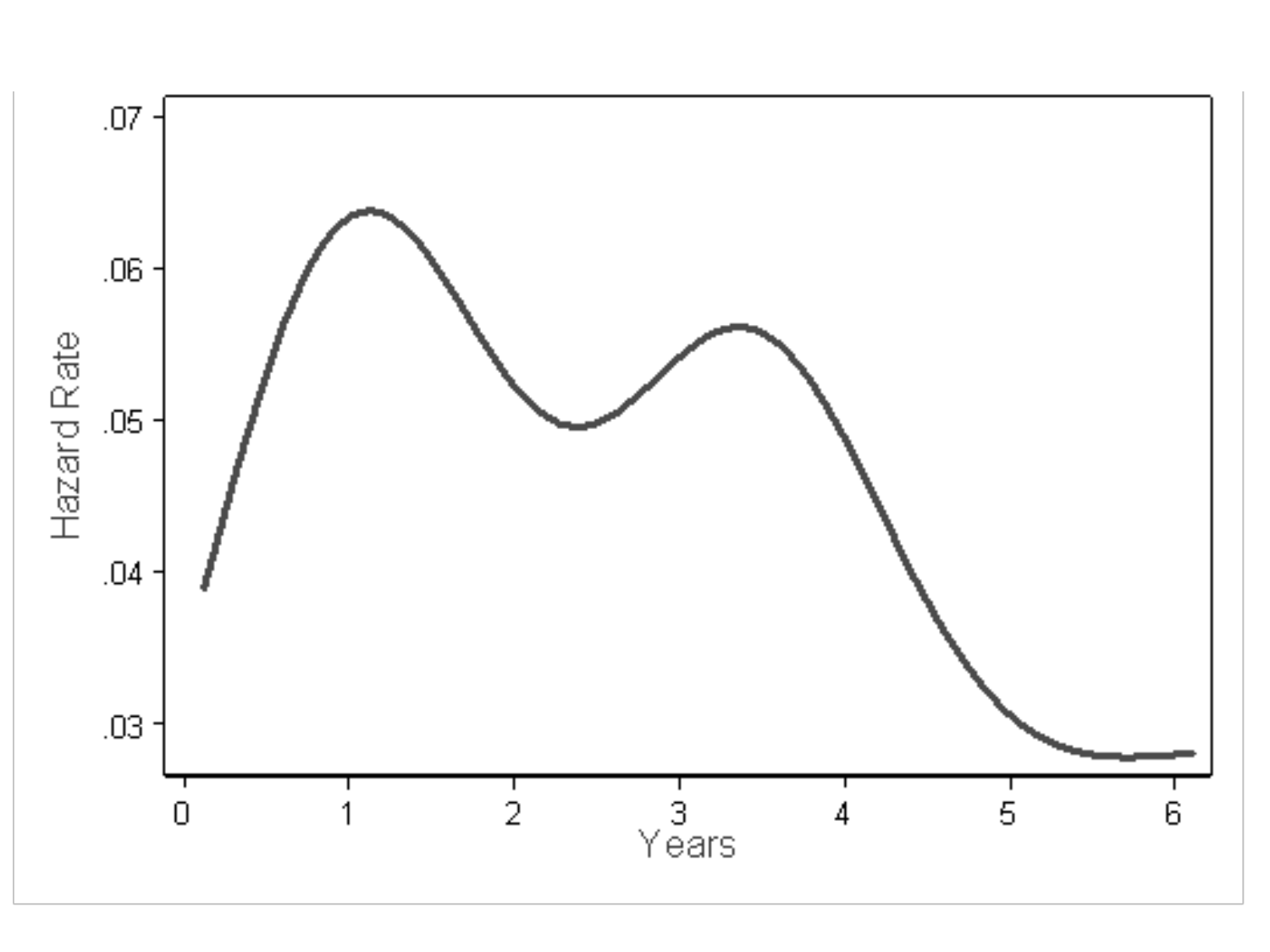

Supplement: Additional file 5: — Relapse Rates of the patients with TNBC according to time ( n =204). (PPTX 103 kb) [file 12885_2015_1977_MOESM5_ESM.pptx]
